# Prion2022-pushing the boundaries

**DOI:** 10.1080/19336896.2023.2175580

**Published:** 2023-02-13

**Authors:** Inga Zerr

**Affiliations:** Department of Neurology, University Medical Center, Georg-August-Universität Göttingen, Göttingen, Germany

Prion2020/22, the world’s largest annual research conference focused on prion and protein-misfolding diseases was held 13–16 September 2022 at the Central Lecture Building (Zentrales Hörsaalgebäude, ZHG) of the Georg-August University of Göttingen (https://prion2022.org). The Göttingen Prion2020 meeting was postponed two times because of the pandemic situation due to COVID-19. The Conference Prion2020/2022 followed a series of international meetings in the field, being held annually since 2004 at various places around the world (https://www.neuroprion.org). The conference provides a platform for discussion for scientists coming from various fields – from clinicians, veterinarians, pathologists, biochemists, neuroscientists and epidemiologists to structural biologists, which leads to a fruitful exchange of knowledge and ideas across disciplines.

Prions are the first known example of transmissible agents that are not bacteria, viruses, fungi, or parasites and are considered as ‘pure’ proteins. These transmissible agents are discussed to be devoid of genetic information. Prions are responsible for degenerative brain diseases like Creutzfeldt-Jakob Disease (CJD) in humans, Bovine Spongiform Encephalopathy (Mad cow disease) in cattle, Scrapie in sheep and goats and Chronic Wasting Disease in cervids. They are a public health problem due to the transmissible nature of the disease and, therefore, demand constant attention from governments and the scientific community.

The term prion was originally introduced by Stanley Prusiner in 1982 to define the peculiar properties of the proteinaceous infectious particle found in Scrapie, a form of spongiform encephalopathy affecting sheep. The same component was subsequently pinpointed as the culprit of a class of infectious, inherited and sporadic human diseases (CJD, Gerstmann–Sträussler–Scheinker syndrome and fatal familial insomnia). Foremost, the term prion underlies the requirement of infectivity, although the spreading in the absence of a nucleic acid was unprecedented, putting the basis for a newfangled field in biology.

The presence of protein deposits is a common pathological hallmark in patients suffering from neurodegenerative conditions and other proteinopathies. In recent years, the compelling presence of insoluble, β-enriched proteinaceous aggregates lead the researchers to speculate that some of these proteins would behave like the prion protein, such as amyloid-β and Tau, the major constituent of Alzheimer’s disease (AD) plaques and tangles, α-synuclein in Parkinson’s disease and others. Since then, prion biology is being increasingly recognized as relevant to the understanding of a subset of neurodegenerative disorders, such as Alzheimer’s and Parkinson’s diseases that affect millions every year. Knowledge obtained from the study of prions and prion diseases might prove to be of enormous interest in the near future, especially since definite therapy is lacking. The understanding of the prion or prion-like mechanisms in neurodegenerative diseases will be important for drug development in the future.

Without neglecting other very important aspects of human and animal prion diseases, the Organizing Committee has therefore decided to open the conference for other themes in the neurodegeneration field. The programme highlighted an update and a discussion of what is known about prion diseases, but also of other neurodegenerative diseases such as Alzheimer’s diseases and synucleinopathies and resulted in lively discussions on common aspects and distinct properties here. The cross-talk beyond the borders of own research facilitated fruitful collaborations between the disciplines and research fields and stimulates the progress in the understanding of the basis of neurodegeneration in prion- and prion-like disorders.

The Prion2020/2022 brought together leading scientists in the field of prion- and prion-like disorders, an emerging field due to recent findings in the context of Alzheimer’s and Parkinson’s diseases, and discussed the latest developments in structural biology, prion propagation, transmission, animal and human diseases. Important topics were the nature of the agents, the properties of aggregation-prone proteins, the risk to human and animal health and emerging therapeutic concepts, which were covered in 14 thematic sessions. One of the sessions and two family foundation meetings were organized by patient/family organizations.

The conference attracted many young scientists at the start and in the early stages of their careers and provided ample opportunities for them to speak and present their work. In total, 44 oral presentations were selected, and 223 posters were presented (https://www.tandfonline.com/doi/full/10.1080/19336896.2022.2091286). The day before the conference, four specific half-day pre-conference workshops on animal diseases, human pathology, structural biology and biomarkers were held.

As a final remark, the first international conference on prion diseases took place in 1995 in Göttingen and was organized at that time by Hans Kretzschmar. In the following years, annual meetings were established. Prion2022 attracted scientists from 27 countries, actively involved early-stage researchers and provided a platform for lively discussions on recent advancements in the research field of neurodegenerative diseases, beyond the borders of the initially established prion concept. The series of successful conferences will hopefully continue. The next meeting is already scheduled for autumn 2023 (https://prion2023.org/)

